# Comparative genomic analysis of driver mutations in matched primary and recurrent meningiomas

**DOI:** 10.18632/oncotarget.26941

**Published:** 2019-05-28

**Authors:** Joshua Loewenstern, John Rutland, Corey Gill, Hanane Arib, Margaret Pain, Melissa Umphlett, Yayoi Kinoshita, Russell McBride, Michael Donovan, Robert Sebra, Joshua Bederson, Mary Fowkes, Raj Shrivastava

**Affiliations:** ^1^ Department of Neurosurgery, Icahn School of Medicine at Mount Sinai, NY, New York, USA; ^2^ Department of Genetics and Genomic Sciences, Icahn School of Medicine at Mount Sinai, NY, New York, USA; ^3^ Department of Pathology, Icahn School of Medicine at Mount Sinai, NY, New York, USA; ^4^ Department of Otolaryngology, Icahn School of Medicine at Mount Sinai, NY, New York, USA

**Keywords:** meningioma, tumor recurrence, genomics, driver mutation, progression-free survival

## Abstract

A significant proportion of low-grade WHO grade I and higher-grade WHO grade II or III meningiomas are at risk to develop post-resection recurrence. Though recent studies investigated genomic alterations within histological subtypes of meningiomas, few have compared genomic profiles of primary meningiomas matched to their recurrences. The present study aimed to identify oncogenic driver mutations that may indicate risk of meningioma recurrence and aggressive clinical course. Seventeen patients treated for low-grade (*n* = 8) or high-grade (*n* = 9) meningioma and underwent both primary and recurrent resection between 2007–2017 were reviewed. Tumor specimens (*n* = 38) underwent genomic sequencing of known oncogenic driver mutations. Primary and recurrent tumors were compared using matched-pair analyses for mutational associations with clinical outcomes including functional status, progression-free survival (PFS) and overall survival (OS). Most common driver mutations included *POLE* and *NF2*. There was no enrichment for any driver mutation from primary to recurrent tumor specimen. *NF2* mutant meningiomas were associated with larger tumor size (8-fold increase), presence of vasogenic edema, and higher mitotic proliferation on univariate and independently on multivariate regression (p’s < 0.05) after controlling for preoperative and tumor features. Tumors with *POLE* driver mutations were associated with decreased functional status at last postoperative follow-up (*p* = 0.022) relative to presentation. Mutation status was not associated with PFS or OS on multivariate Cox regression, but rather with grade of resection (*p* = 0.046) for PFS. While primary and recurrent tumors exhibited similar driver mutations within patients, the identification of driver mutations associated with clinical outcomes is crucial for guiding potential targeted treatments in recurrent meningiomas.

## INTRODUCTION

Meningiomas are the most common primary brain tumor, accounting for approximately 34% of all primary intracranial neoplasms. While the majority of meningiomas are classified as WHO grade I and are benign, approximately 20% are grade II (atypical) and grade III (anaplastic). Grade II meningiomas account for 5–15% of all meningiomas, can exhibit atypical histological features that include prominent nucleoli, hypercellularity, necrosis, high nuclear-to-cytoplasmic ratio, or brain invasion [1]. Grade II meningiomas are more likely to grow more rapidly than benign meningiomas and have a greater likelihood of recurring following gross total resection. Adjuvant radiation therapy (RT) is often prescribed in the case of subtotal resection of Grade II meningiomas. Grade III meningiomas comprise the smallest proportion of cases, about 1–3%, and have poor oncological outcomes. Grade III meningiomas grow at a faster rate than both benign and atypical tumors and are more likely to invade brain tissue, metastasize to other organs, and recur than the Grades I and II meningiomas [2, 3].

Diagnosis of grade II and III meningiomas can manifest heterogeneous clinical outcomes [2, 4]. Reports of 5-year progression free survival rates for combined Grades II and III meningiomas after radical resection range from 20%–50% [2, 4], reinforcing the highly variable long-term results for these tumors. Current markers of improved prognosis are poorly defined in the literature and cannot account fully for this divergence in individual clinical behaviors of atypical and malignant meningiomas, making treatment prediction and the decision for aggressive multimodal therapy difficult. Indeed, the current WHO grading system relies on histopathological features and is often imprecise and insensitive to predict long-term oncologic outcomes such as recurrence and survival.

Recently, there has been increased interest in elucidating how genomic alterations may inform treatment stratification in intracranial tumors [5, 6]. There is a critical need to better understand genomic alterations that are associated disease outcomes such as tumor recurrence, response to radiotherapy, and survival. Meningiomas that are incompletely resected due to anatomical location or are grade II or III have increased risk of recurrence. In these cases, adjuvant radiotherapy is commonly used to target the surgical bed and has been shown to improve local control [7] and survival [8, 9]. However, the utility of the wait-and-see strategy for treating meningioma is debated and is currently being investigated by the NCT03180268 and ROAM/EORTC-1308 trials [10, 11]. Therefore, identifying genomic markers that may be associated with treatment resistance or contribute to meningioma recurrence is important.

Prior studies have established compelling evidence for the role of genomic characterization in meningioma pathogenesis. The tumor suppressor gene, neurofibromin 2 (*NF2*), is well studied in the context of meningioma. Mutations in *NF2* have been associated with the development of benign intracranial tumors, including schwannomas and meningiomas [12, 13]. Recently, alterations in other oncogenic genes such as *TRAF7*, *AKT1*, *KLF4*, *PIK3CA*, *SMO,* and *DMD* have also been implicated in meningioma etiology [14, 15]. In their review of 553 meningiomas, Yuzawa et al reported *NF2*, *TRAF7*, *AKT1*, *KLF4, PIK3CA*, and *SMO* mutations in 55, 20, 9, 9, 4.5, and 3% of tumors [16].

Others have sought to identify genomic determinates of meningioma aggression. Bi et al observed that *PIK3CA* and Hedgehog pathway mutations were indicative of low-grade meningiomas, whereas *NF2* mutation and widespread genomic alterations were characteristic of high-grade tumors [17, 18]. Additionally, the promoter region of the gene that encodes the reverse transcriptase component of telomerase (*TERT*), has also been implicated in meningioma behavior.

Despite these findings, there has been limited success in identifying how mutation status may influence treatment outcomes. Further, only one prior study has compared mutation status from primary meningioma to subsequent recurrence(s) [19]. The focus of this study aims to characterize the genomic profiles of matched primary and recurrent meningiomas in 17 patients and associate mutation status with critical treatment outcomes including recurrence, functionality, and survival.

## RESULTS

### Patient and tumor characteristics

**Figure 1:**
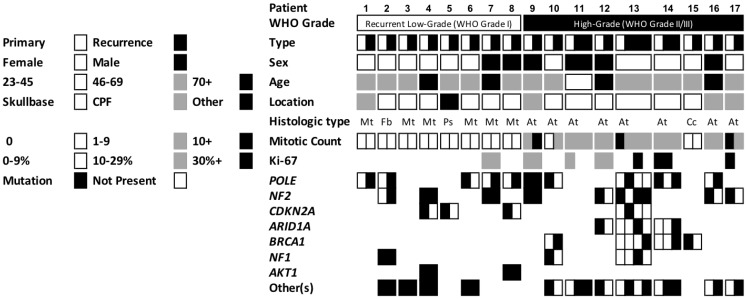
Demographics, tumor features, and mutation status of the cohort of 17 patients with matched targeted sequenced primary and recurrent meningioma. The seven most common driver mutations of the cohort are depicted. Each tumor was analyzed separately for presence of driver mutations. Abbreviations: CPF, convexity, parasagittal, or falcine; other, spinal; Mt, meningothelial; Fb, fibroblastic; Ps, psammomatous; At, atypical; Cc, clear cell.

Demographic and tumor features along with mutation status among paired primary and recurrent meningiomas are depicted in Figure 1. The overall sample consisted of a majority of females (65%) with an average age of 61 years (SD = 11.6 years) on initial presentation. Patients did not differ in their preoperative functional status from primary to recurrent tumor (*p* = 0.13). Most meningiomas were located in skull base regions (71%), followed by convexity, parasagittal, or falcine regions (24%), and 1 was located in a spinal region (see Table 1). Median tumor volume was 3.5 cm^3^ (IQR = 0.9–11.8 cm^3^) and was significantly larger for primary tumors compared to their matched recurrences (4.7 vs. 1.7 cm^3^, *p* = 0.019). A minority of primary and recurrent tumors presented with associated vasogenic edema (29 vs. 24%, *p* > 0.99). The majority of tumors were completely resected (84%), which did not differ from primary to recurrence (*p* = 0.34).

**Table 1:** Preoperative and tumor characteristics of matched primary tumors to first recurrence (17 pairs = 34 tumors)

	Primary (*n* = 17)	Recurrence (*n* = 17)	*P*-value
Variable	*N* (%) or Median (IQR)	*N* (%) or Median (IQR)	
***Clinical Features***			
Preop Functional Status (KPS)	70 (70–80)	80 (70–90)	0.13
Tumor Location			>0.99
*Skull base*	12 (70.6)	12 (70.6)	
*CPF*	4 (23.5)	4 (23.5)	
*Other*	1 (5.9)	1 (5.9)	
Tumor Volume (cm^3^)	4.7 (1.1–31.2)	1.7 (0.6–7.3)	0.019
Presence of Vasogenic Edema			>0.99
*Yes*	5 (29.4)	4 (23.5)	
*No*	12 (70.6)	13 (76.5)	
Simpson Grade			0.34
*Gross Total (1–3)*	13 (76.5)	16 (94.1)	
*Subtotal (4–5)*	4 (23.5)	1 (5.9)	
Presence of Brain Invasion			>0.99
*Yes*	2 (11.8)	2 (11.8)	
*No*	15 (88.2)	15 (88.2)	
WHO Grade			>0.99
I	8 (47.1)	8 (47.1)	
II	9 (52.9)	9 (52.9)	
III	0 (0.0)	0 (0.0)	
Mitotic Index [Mean (SD)]	2.47 (4.6)	2.29 (3.3)	0.75
≥4 per 10 HPF	5 (29.4)	5 (29.4)	>0.99
Ki67/MIB-1 (%)	22.5 (20–28.8)	20 (16.3–27.5)	0.77
***Genomics***			
Multiple (≥2) Driver Mutations	8 (47.1)	9 (52.9)	>0.99
Total Driver Mutations	1 (1–2)	1 (1–2)	>0.99
***Outcomes***			
Postop Functional Status (KPS)	80 (75–90)	80 (70–95)	0.36
*Decreased KPS relative to Preop*	2 (11.8)	3 (17.6)	0.48
Functional Status at last follow-up (KPS)	80 (65–90)	80 (65–90)	>0.99
*Decreased KPS relative to Preop*	5 (29.4)	6 (35.3)	>0.99

Abbreviations: KPS, Karnofsky Perfornance Status; CPF, convexity, parasagittal, or falcine.

^*^Ki-67/MIB-1 available for 11 (32.4%) tumors.

Values represent *N* (%) or Median (IQR), as appropriate, unless otherwise specified. Compared matched pairs with McNemar’s test for paired proportions or Wilcoxon signed-rank test.

Eight (47%) patients had recurrent WHO grade I meningiomas and 9 (53%) had recurrent atypical WHO grade II meningiomas. No matched pairs of anaplastic WHO grade III meningiomas were encountered in our cohort. Of the WHO grade I meningiomas, 12 (75%) were meningothelial subtype, 2 fibroblastic, and 2 psammomatous. Within the WHO grade II specimens, the majority (91%) were atypical subtype and 2 were clear cell. On histopathological examination, brain invasion was noted in a minority of tumors (13%). Mean mitotic index did not differ from primary tumor to recurrence (2.47 vs. 2.29 per 10 HPF, *p* = 0.75). Ki67 labeling similarly did not statistically differ for those cases with Ki67 labeling (32%).

### Genomic profiles

Most common driver mutations are depicted by matched pairs in Figure 1. *NF2* and *POLE* driver mutations were each present in 13 (34%) and 12 (32%) of the 38 total primary and recurrent tumor samples, respectively. *CDKN2A*, *ARID1A*, *BRCA1*, *NF1*, and *AKT1* were also commonly identified in meningioma samples across the cohort. Other driver mutations identified in one or two tumors (30 other genes identified, see Supplementary Figure 1) included *NOTCH3*, *SMO*, *PIK3CA*, and *BRCA2*. Of note, no driver mutations in the paired cohort were identified for *TERT*, *SMARCB1*, or *BAP1*. There were no differences by presence of a specific driver mutation in regard to patient sex, location of tumor, or grade of resection. Patients with an *NF2* mutant tumor were on average older than those without the mutation (66.5 vs. 56.8 years, *p* = 0.010). There was also no difference between primary tumors and their recurrences regarding tumors with multiple driver mutations (47 vs. 53%, *p* > 0.99) or median number of total driver mutations (*p* > 0.99, Table 1).

### Associations with tumor features

**Figure 2:**
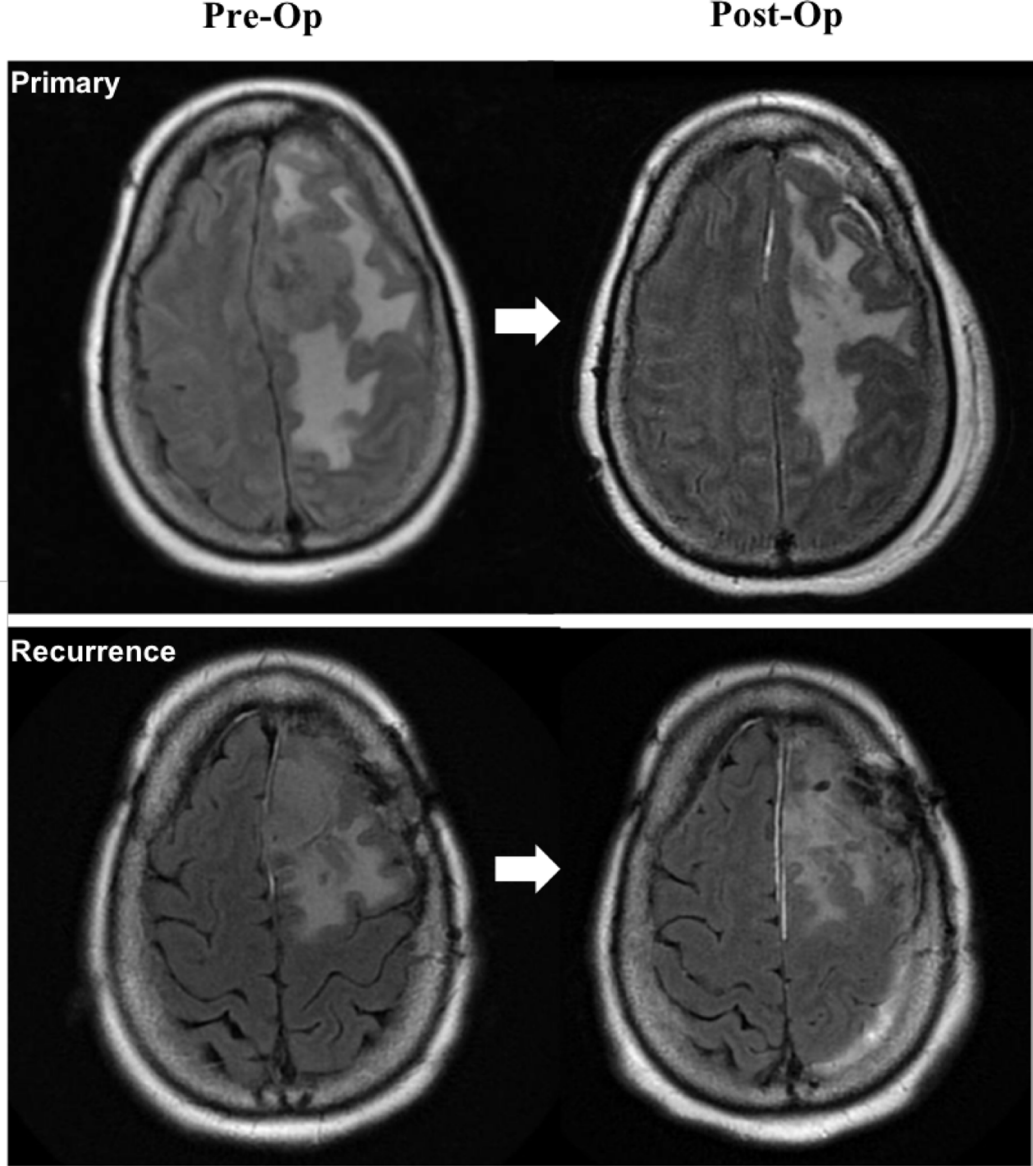
T2 FLAIR magnetic resonance (MR) imaging of a representative patient with an *NF2* mutation positive WHO grade II meningioma (frameshift deletion at locus chr22:30074229) showing large tumor volume with vasogenic edema for the primary tumor (*top*) and a recurrence (*bottom*). Left images depict preoperative MR imaging and right images are postoperative MR imaging. Recurrent meningioma was found to have an *ARID1A* nonsense mutation at locus chr1:27101099 in addition to the *NF2* frameshift deletion.

Several driver mutations were found to be associated with specific tumor features. *AKT1* mutations were found in more WHO grade I tumors than higher grade meningiomas (*p* = 0.020). Additionally, *NF2* mutation status was associated with several tumor features. A majority (63.6%) of tumors presenting with vasogenic edema on preoperative imaging had an *NF2* driver mutation compared to 22.2% of tumors without edema (*p* = 0.020). On multivariate regression, the presence of an *NF2* mutation was found to be an independent predictor of larger tumor volume (Beta = 19.8, *p* = 0.008, see Table 2), controlling for other preoperative patient characteristics and tumor features. Median tumor volume with the mutation was 14.3 cm^3^ (IQR = 3.7–47.2 cm^3^) relative to 1.7 cm^3^ (IQR = 0.9–4.9 cm^3^) without the mutation (*p* = 0.021), an over eight-fold increase in median tumor size. Figure 2 depicts a representative patient with a large primary tumor harboring an *NF2* driver mutation with vasogenic edema prior to initial resection compared with the patient’s recurrent tumor also presenting with substantial vasogenic edema. Moreover, presence of an *NF2* mutation was found to be an independent predictor of mitotic index on multivariate regression analysis (beta=3.0, *p* = 0.006, Table 2). Mean mitotic index for *NF2* mutation positive tumors was 4.7 per 10 HPF (SD = 5.4) compared to 1.5 (SD=2.3) without the mutation (*p* = 0.014).

**Table 2:** Multivariate regression analyses on tumor volume and mitotic index and Cox multivariate regression analyses on progression-free survival (PFS) and overall survival (OS)

Characteristic	Multivariate on Tumor Volume			
	**Beta**	**SE**	**95% CI**	***P*-value**
***NF2* mutation**	19.8	7.0	5.6–34.0	0.008
	**Multivariate on Mitotic Index**			
	**Beta**	**SE**	**95% CI**	***P*-value**
**WHO Grade**	4.3	1.0	2.3–6.3	<0.001
***NF2* mutation**	3.0	1.0	0.9–5.0	0.006
	**Cox Multivariate on Progression-Free Survival**			
	**HR**	**95% CI**	***P*-value**	
**Simpson grade of resection**	3.88	1.02–14.7	0.046	
	**Cox Multivariate on Overall Survival**			
	**HR**	**95% CI**	***P*-value**	
**Preop Functional Status (KPS)**	0.83	0.66–1.05	0.095	

Abbreviations: SE, standard error; HR, hazard ratio; CI, confidence interval; KPS, Karnofsky Performance Status.

Preoperative clinical, demographic, and tumor features were entered into each model. Significant variables are shown only.

### Associations with clinical outcomes

The median time for patients to develop a meningioma recurrence after primary tumor resection was 7.0 months (IQR=3.5 to 18.0 months). The present analysis did not find any association with time to recurrence for any specific driver mutation. The only factor found to be associated with timing of recurrence on multivariate Cox regression analysis, with preoperative patient characteristics and tumor features entered as covariates, was Simpson grade of resection at primary resection such that tumors with a subtotal resection were more likely to progress in less time than those with a complete resection (HR=3.88, *p* = 0.046, see Table 2).

At initial postoperative follow-up and last follow-up examination, patients did not differ in median functional status from their primary meningioma to their recurrence (*p* = 0.36 and >0.99, respectively, see Table 1). Further, there was no difference within matched pairs in the proportion of patients that decreased in functional status at their initial follow-up or last follow-up relative to preoperative functional status (*p* = 0.48 and >0.99, respectively).

Half of the patients underwent adjuvant RT after either primary or recurrent tumor resection. Two (22%) radiation treatments occurred shortly after primary resection, while the remaining 7 patients (78%) were not irradiated until after the recurrent tumor resection. Use of adjuvant RT correlated with wildtype *NF2* status (*p* = 0.020). As of the completion of this study, 12 (71%) patients were alive, 3 (18%) were dead, and 2 were lost to follow-up. The overall median observation time for patients was 49.5 months (IQR=34.1 to 81.2 months). The present study did not find any significant independent predictors of risk for mortality on Cox regression analysis (Table 2).

## DISCUSSION

In a single-center analysis of matched tumor specimens of 17 patients who underwent primary and recurrent meningioma resection during over a 10-year period, we aimed to compare mutation status in determining significant predictors of tumor recurrence, survival, and other clinical outcomes. Though matched primary and recurrent tumors shared similar driver mutations as expected, genomic analysis revealed key associations among mutations and tumor features and clinical outcomes, particularly involving the *NF2* gene.

### Preoperative presentation and tumor features

Matched primary and recurrent meningiomas differed only in tumor size among preoperative and tumor features (Table 1), which is likely due to serial follow-up imaging and neurologic exams aimed to detect recurrent tumors before presentation of clinical sequelae. However, there were notably no differences on the whole between primary and recurrent tumors in regard to affecting presenting functional status or specific tumor features including vasogenic edema, brain invasion, or proliferation index. Given that WHO grade remained constant between primary tumor and recurrence, it is not surprising that tumor proliferation and behavior were similar, though a larger sample may better characterize such differences [1].

After targeted sequencing for clinically actionable driver mutations, the most common identified were *NF2* and *POLE*. A total of 13 primary or recurrent specimens (34%) were *NF2* mutation positive, which is consistent with prior genetic investigations that have cited sporadic *NF2* mutations in up to half of studied meningiomas [16, 17, 20]. For example, Yuzawa et al. reported an NF2 mutation in 55% of meningiomas reviewed in a large sample while Bi et al. similarly found NF2 mutations in 53% of their meningioma sample [16, 17]. *POLE* variants, which were also present in 12 tumors (32%) in the sample and encode for the catalytic subunit of DNA polymerase epsilon, have not commonly been identified in prior meningioma genomic studies, but have been implicated in syndromes related to neurofibromatosis type 1 [21]. Other common driver mutations in our cohort such as *CDKN2A*, *ARID1A*, *BRCA1*, *NF1*, *AKT1*, *SMO*, *PIK3CA* (see Figure 1 and Supplementary Figure 1) have similarly been noted in various prior sequencing studies of meningiomas [13, 16, 17, 22–26].

Of note, other common genetic variants identified in prior studies including *TERT*, *SMARCB1*, and *BAP1* were examined as part of the panel, but not identified as driver mutations in the current sample cohort. While the present analysis did not identify driver mutations within the *TERT* gene, our technique did not evaluate the *TERT* promoter region which, through effects on telomerase expression, has been linked to meningioma recurrence and survival in a number of past studies [15, 26–32]. However, Juratli et al. has also demonstrated variant rearrangements within the *TERT* genetic region within a sample of treatment-resistant high grade meningiomas, which was not replicated in our sample of matched tumors [27]. *SMARCB1* mutations, involved in gene expression regulation and also located in close proximity to *NF2* on chromosome 22, have also been identified in particularly atypical meningiomas in prior work, but were not identified in our matched tumor pairs [33–35]. Similarly, *TRAF7* and *KLF4* mutations have been linked to meningioma [14, 16, 33], but were not assessed by the panel of the present study. As *TRAF7* mutations were found in up to a quarter of meningiomas in prior studies, particularly in secretory meningiomas [14, 33], such mutations would likely have been present in our matched tumor pairs. Recently, *DMD* inactivation was linked to risk of mortality [15], but similarly was not assessed in the present clinically actionable driver mutation-targeted study.

Further, we did not find any differences between primary and recurrent tumors by specific driver mutation in regard to baseline characteristics (e.g., sex, location, grade of resection), though previous work has noted, for example, a tendency for non-*NF2* variant tumors such as *AKT1* and *SMO* to develop in skull base regions relative to CPF regions [25, 33, 36]. However, several driver mutations were associated with specific tumor features including WHO grade. For example, tumors with an *AKT1* driver mutation tended to be low-grade.

### Role of NF2 mutations

**Figure 3:**
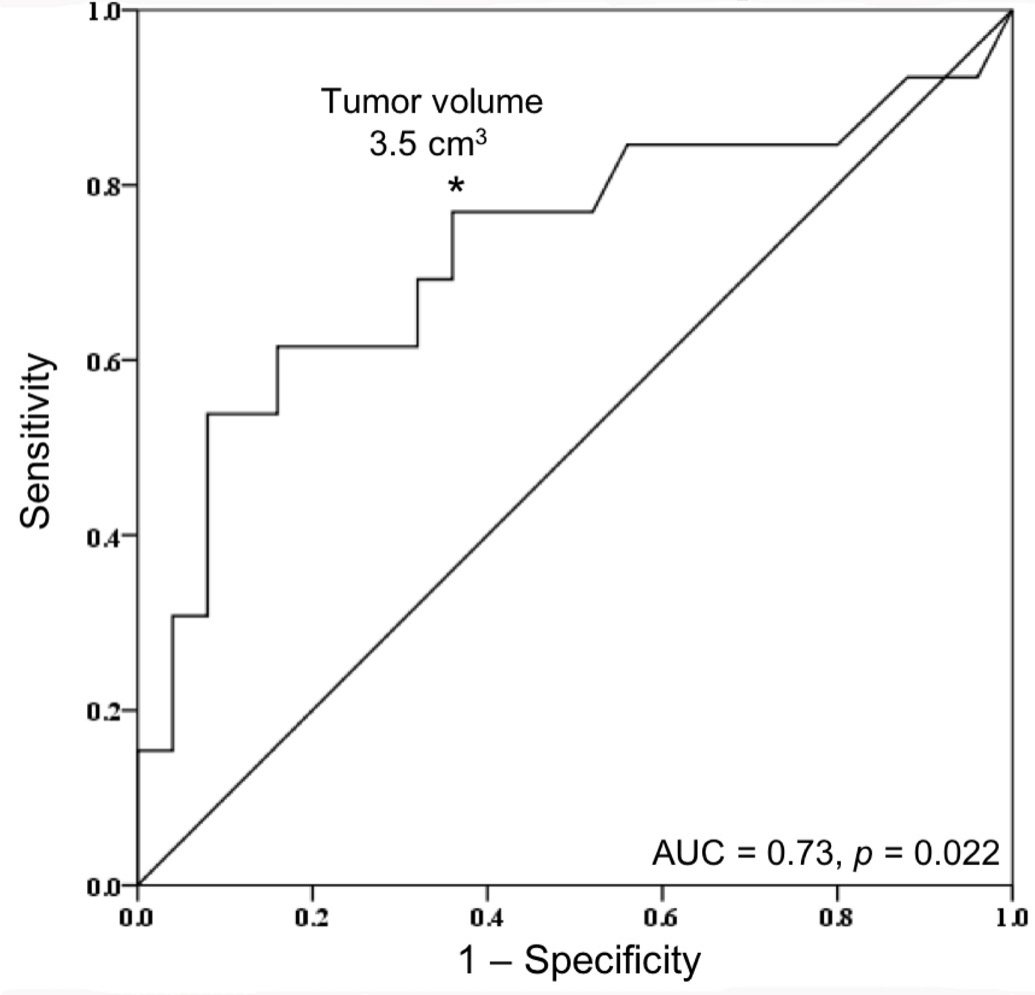
Receiver operating characteristic (ROC) curve of tumor volume by *NF2* mutational status. Area under the curve (AUC) was equal to 0.73 (*p* = 0.022). ^*^At a tumor volume of greater than or equal to 3.5 cm^3^, sensitivity and specificity for predicting NF2 mutational status was 76.9% and 63.0%, respectively. Positive predictive value (PPV) and negative predictive value (NPV) was 52.6% and 84.2%, respectively.

*NF2* mutations were found to be significantly associated with several tumor features and patient characteristics. Patients with *NF2* mutant meningiomas were on average 10 years older than patients without an *NF2* mutation, suggesting that *NF2*-driven meningiomas may display a slower growing, more indolent disease course. Additionally, *NF2* mutation was significantly associated with larger tumor volume. This may also indicate that meningiomas driven by *NF2* mutations are slower growing and only become symptomatic when they become very large and cause mass effect on surrounding structures. Receiver operating characteristic (ROC) curve depicting tumor volume by *NF2* mutational status is shown in Figure 3. At a tumor volume of greater than or equal to 3.5 cm^3^ on preoperative MRI, volume predicted *NF2* mutational status with a sensitivity of 76.9%, specificity of 63.0%, positive predictive value (PPV) of 50.0%, and negative predictive value (NPV) of 85.0%. Thus, finding a small tumor volume on preoperative imaging may be a fair indicator of non-*NF2* mutational status, but larger cohorts are needed to support this finding.

Tumor volume may have also mediated the increased vasogenic edema observed in tumors with *NF2* mutation. Vasogenic cerebral edema is a type of extracellular edema that occurs when the tight junctions of the blood brain barrier is disrupted, and results in leakage of fluids from capillaries into the interstitial brain compartment. Morimoto et al. reported significant correlation between tumor volume and degree of peritumoral edema in intracranial meningiomas, suggesting that larger tumors may cause greater edema [37].

Additionally, merlin, the *NF2*-encoded protein, may play a role in stabilizing intercellular junctions in the blood brain barrier, so vasogenic edema may be mediated through absence of this gene product [32]. *NF2* is a tumor suppressor gene that is located on the long arm of chromosome 22q12, and was the first single-gene mutation to be linked with risk of developing meningioma [38, 39]. Merlin, which is responsible for a number of inhibitory cellular functions including Rac-PAK pathway suppression [40, 41], inhibition of the PI3K-Akt pathway [42], and suppression of the EGFR-Ras-ERK pathway [43]. Merlin’s inhibitory functions promote contact inhibition and suppress mitotic signaling at cellular junctions [40]. While *NF2* mutation-driven meningiomas have been shown to differ from non-*NF2* mutation-driven meningiomas with regard to histological subtype and intracranial location [44], the phenotypic and oncological features of meningiomas driven by *NF2* mutation status remain inadequately described.

Moreover, the findings also suggest that *NF2*-driven meningiomas may exhibit a more indolent course than non-*NF2*-driven meningiomas. Because subtotal resection is the most common indication for postoperative RT, and tumors with *NF2* mutations received less adjuvant RT, it is also possible that *NF2* mutation-driven meningiomas are easier to resect, due to surgical access of their specific intracranial location. In the present study, 8% of meningiomas with *NF2* mutations were subtotally resected compared with 19% of non-*NF2*-driven meningiomas. While this effect was not significant, larger studies are needed to determine whether *NF2* mutation plays a role in extent of resection.

Taken together, these findings suggest that *NF2* mutant meningiomas tend to be slower growing, more indolent tumors than meningiomas harboring other genomic alterations. Interestingly, the incidence of *NF2* mutations is fairly proportional among WHO grades. This finding suggests that *NF2* mutation is involved in an early initiation event in meningioma tumorigenesis as opposed to tumor progression [45]. However, genomic alterations in atypical and anaplastic meningiomas are multifactorial, involving numerous genetic changes, and the role of *NF2* in tumor progression requires further investigation. Implications of *NF2* mutations significantly influencing meningioma progression have prompted clinical trial investigations of inhibition of focal adhesion kinase, thought to be associated with *NF2*/merlin-mediated tumor growth [11, 22]. Execution of high throughput genomic analysis in large multi-center studies are needed to further elucidate the role of *NF2* within the complex phenotypic manifestations of meningiomas.

### Genomic associations with clinical outcomes

**Figure 4:**
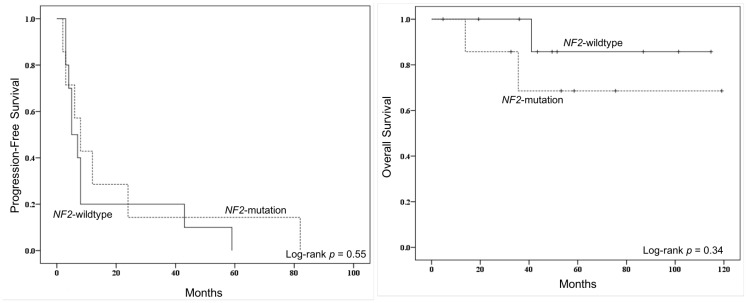
Kaplan–Meier progression-free survival (PFS) curve (*left*) and overall survival (OS) curve (*right*) for comparisons by NF2 mutational status. Comparisons were made by log-rank tests.

Among the recurrent meningiomas in this matched cohort, we did not find driver mutations to be associated with time to recurrence on univariate analysis, even though the median time to recurrence was 7.0 months, consistent with prior studies of meningioma recurrence. On multivariate Cox regression analysis, the only independent predictor of PFS was grade of resection such that a subtotal resection was associated with a higher risk of progression in relatively less time than a complete resection (see Table 2). Prior genomic studies, however, have linked the presence of an *NF2* mutation to increased recurrence relative to other variant drivers [16, 17]. For example, Yuzawa and colleagues reported 23% of tumors with an *NF2* mutation had a recurrence within the study follow-up period [16]. Moreover, *NF2* mutations were recently shown to contribute to a decreased PFS in a sample of WHO grade I and II meningiomas [20]. In our sample, primary tumors with an *NF2* mutation recurred in a mean of 14.5 months relative to 19.6 months for those without such a mutation, but this was not significant given the underpowered sample (*p* = 0.74). Kaplan-Meier plot of PFS for patients with and without an *NF2* mutation is depicted in Figure 4. Though not investigated in this study, *TERT* promoter mutations have been demonstrated in prior work to be associated with unfavorable PFS across low- and high-grade meningioma [28, 29, 31]. Taken together, the findings suggest drivers for recurrence and tumor behavior are likely present in the primary tumor and are less likely, in the majority of cases, acquired mutations in residual tumor cells post-resection. However, future studies should continue to investigate the genetic associations with tumor recurrence and, specifically, tumor subtypes in which acquired mutations may have a larger role in driving recurrence.

In regard to postoperative functional status, patients possessing primary or recurrent tumors with *POLE* driver mutations had decreased functional status post-resection compared to those patients without such genomic alterations. First, *POLE*, along with *NF2*, was the most common encountered mutation in the matched pairs and was associated with decreased functionality at last follow-up. Though *POLE* mutations in higher-grade meningioma have only recently been reported in prior work [46], *POLE* mutations have been linked to worse prognosis in other cancers such as in particular subgroups of colorectal and endometrial cancer patients [47].

Lastly, on analysis of mortality risk, the present analysis did not uncover an association with any driver mutation with survival, including presence of an *NF2* mutation (see Table 2 and Figure 4). Patients presenting with a severely symptomatic meningioma tend to have poorer outcomes post-resection, including a potential increased risk for mortality [48, 49]. While not identified within our matched tumor sample, prior genetics work have linked *CDK2NA* mutations to decreased survival in anaplastic meningioma [50], and *TERT* promoter mutations to decreased OS in both higher grade meningioma [29] and across tumor grades [31]. Though the median follow-up time was over 4 years after initial resection, a larger cohort of patients with a longer follow-up time may reveal genetic associations with mortality risk.

### Limitations

There were several limitations of the present study including its retrospective nature and associated biases in design. First, we limited our analysis to include only patients with surgical resection of both a primary and recurrent meningioma at our medical center. Second, we did not perform sequencing to assess germline status from normal samples for each patient to control for germline variants. We therefore acknowledge that some of our identified variants may be germline in nature and not specific to the tumor. Third, our study utilized a targeted panel of oncogenic genes associated with cancer and did not include whole-exome or whole-genome sequencing. As such we were note able to assess gene status of several known meningioma-associated mutations nor the *TERT* promoter mutations. Fourth, we performed sequencing of only one specimen for each surgical resection, and thus we were note able to account for genomic heterogeneity that may underlie high-grade meningiomas. Such a limitation can likely account for why certain clonal driver mutations were not identified in both primary and recurrent specimens. Overall, the analysis was bounded by several clinical limitations and the interpretations were made from the best clinical information available.

## MATERIALS AND METHODS

### Patient cohort

We performed a search of our institutional database to identify patients, with available archived formalin fixed paraffin-embedded specimens (FFPE), who presented with a primary meningioma for resection and subsequently had a tumor recurrence additionally resected during a 10-year period from 2007 to 2017. The resulting cohort consisted of 17 patients with matched primary and recurrent tumor samples (see Figure 1 and Table 1). Three patients had more than two resections during this period. Patients who underwent a primary meningioma resection without subsequent recurrence during this period or underwent a recurrent meningioma resection without having received the primary resection at our medical center were, therefore, excluded from this study. Tumor volume was calculated on preoperative MRI by ellipsoid approximation from maximum cross-sectional diameters. Postoperative clinical outcomes measured consisted of functional status, as measured by Karnofsky Performance Status (KPS) score, at initial postoperative and last follow-up, timing of recurrence, and overall survival. The study was approved by the medical center’s Institutional Review Board and informed consent was waived.

### Pathological review

Tumor specimens were reviewed by experienced neuropathologists for tumor grade based on WHO guidelines. Histologic features including presence of brain invasion and mitotic index were assessed. Mitoses were counted per 10 high-power field (HPF) in the area of most prominent mitotic activity. Brain invasion was determined by presence of irregular tumor projections into brain parenchyma or separate islands of tumor surrounded by brain parenchyma without an intervening layer of leptomeninges. For some cases, Ki67 proliferation index was determined at time of initial pathologic review.

### Targeted next-generation genomic sequencing

DNA extraction from FFPE tissues was performed using Maxwell FFPE Plus DNA Purification Kit (Promega). Approximately 50 μm thickness of tissue was used for each extraction. DNA tissue libraries were generated using the Ion AmpliSeq Oncomine Comprehensive research panel versions 2.0 and 3.0 as described previously (https://www.thermofisher.com/us/en/home/clinical/preclinical-companion-diagnostic-development/oncomine-oncology/oncomine-cancer-research-panel-workflow.html) [51]. Sequencing data analysis was performed using Torrent Suite (versions 5.6.0. and 5.8.0.) and Ion Reporter (versions 5.2, 5.6, and 5.8).

### Statistics

Comparisons of matched primary tumor and first recurrence were performed using McNemar’s test for paired proportions and Wilcoxon signed-rank tests, as appropriate. Multivariate stepwise regression analyses were performed on tumor features including tumor volume and mitotic index. Preoperative clinical characteristics and treatment features were entered as covariates. Further, multivariate Cox regression analyses on progression-free survival (PFS) and overall survival (OS) were similarly performed. Survival estimates were compared through the Kaplan–Meier method and evaluated with log-rank tests. Results of multivariate analyses were summarized using beta-coefficients (B) or hazard ratios (HR) and 95% confidence intervals (CI) as appropriate. Analyses were performed using a standard statistical package SPSS (v22.0, IBM, Armonk, NY) and a *p*-value < 0.05 was considered statistically significant.

## CONCLUSIONS

Within a large panel of clinically actionable driver mutations, matched primary and recurrent meningiomas exhibited similar genomic alterations, most commonly in *NF2* and *POLE* genes. *NF2* mutational status was associated with tumor features including size, vasogenic edema, and mitotic proliferation. These findings help to inform how targeted precision therapies may play a role in the treatment of recurrent meningiomas and identify patients at risk for poorer clinical outcomes.

## SUPPLEMENTARY MATERIALS


