# Supplementary material for: Comparative genomic analysis of driver mutations in matched primary and recurrent meningiomas
Source: Oncotarget. 2019 May 28;10(37):3506–17. doi: 10.18632/oncotarget.26941 (PMC6544407; doi:10.18632/oncotarget.26941)
Supplement: Supplementary file 1 [file oncotarget-10-3506-s001.pdf]

Comparative genomic analysis of driver mutations in matched primary and recurrent meningiomas

SUPPLEMENTARY MATERIALS

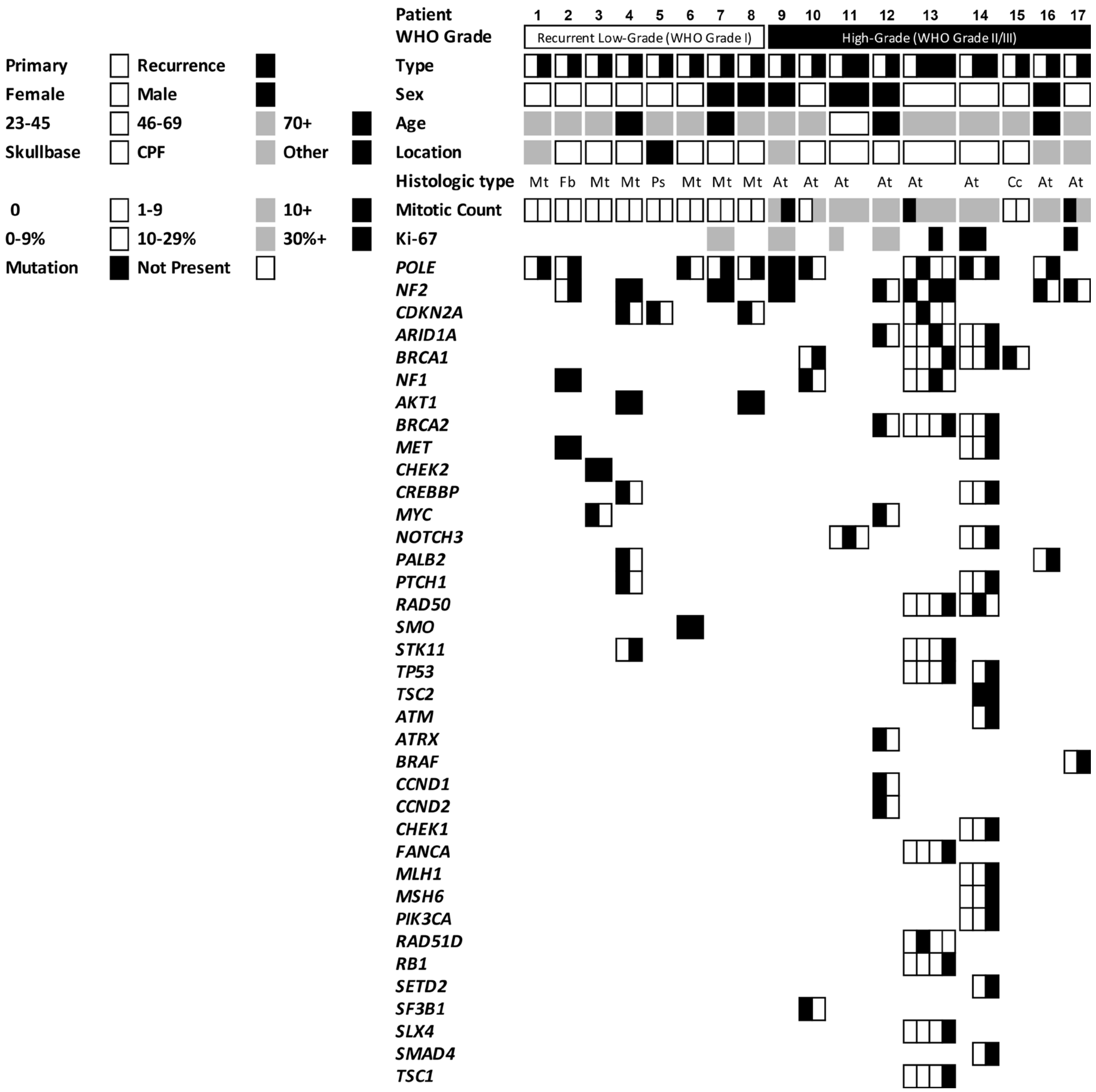

Supplementary Figure 1: Demographics, tumor features, and mutation status of the cohort of 17 patients with matched targeted sequenced primary and recurrent meningioma. All driver mutations of the cohort are depicted. Each tumor was analyzed separately for presence of driver mutations. Tumor location: CPF = convexity, parasagittal, or falcine; other = spinal. Mt = meningothelial; Fb = fibroblastic; Ps = psammomatous; At = atypical; Cc = clear cell.
